# Activation of the sweet taste receptor T1R3 by sucralose attenuates VEGF-induced vasculogenesis in a cell model of the retinal microvascular endothelium

**DOI:** 10.1007/s00417-018-4157-8

**Published:** 2018-10-23

**Authors:** Polina Lizunkova, Emmanuella Enuwosa, Havovi Chichger

**Affiliations:** Biomedical Research Group, Department of Biomedical and Forensic Sciences, Anglia Ruskin University, East Road, Cambridge, CB1 1PT UK

**Keywords:** Vasculogenesis, Diabetic retinopathy, Sweet taste receptors, Artificial sweeteners, Endothelium

## Abstract

**Background:**

One of the most prevalent microvascular complications for patients with diabetes is diabetic retinopathy (DR) associated with increased retinal endothelial blood vessel formation. Treatments to reduce vascularisation in the retinal endothelium are linked to improved sight in patients with DR. Recently, we have demonstrated the novel protective role of the artificial sweetener, sucralose, and the sweet taste receptor, T1R3, in the pulmonary endothelium to reduce vascular leak. In the present study, we examined the role of sucralose and sweet taste receptors on vasculogenic processes (proliferation, migration, adhesion and tube formation) in a cell model of the retinal endothelium.

**Methods:**

We exposed human retinal microvascular endothelial cells (RMVEC) to VEGF as an in vitro model of DR in the presence and absence of T1R3 agonist sucralose.

**Results:**

In RMVEC, we observed increased VEGF-induced cell proliferation, migration, adhesion and tube formation, which was significantly attenuated by exposure to the artificial sweetener sucralose. Following siRNA knockdown of the sweet taste receptor, T1R3, but not T1R2, the protective effect of sucralose on VEGF-induced RMVEC vasculogenic processes was blocked. We further demonstrate that sucralose attenuates VEGF-induced Akt phosphorylation to protect the retinal microvasculature.

**Conclusion:**

These studies are the first to demonstrate a protective effect of an artificial sweetener, through the sweet taste receptor T1R3, on VEGF-induced vasculogenesis in a retinal microvascular endothelial cell line.

**Electronic supplementary material:**

The online version of this article (10.1007/s00417-018-4157-8) contains supplementary material, which is available to authorized users.

## Introduction

Worldwide, there are currently 425 million people estimated to suffer from type 1 and 2 diabetes, which results in a yearly healthcare expenditure of 727 billion USD. By 2045, the number of sufferers is predicted to increase to 693 million, which will further increase the economic burden of the disease [1]. One of the most common and debilitating complications associated with diabetes is the development of diabetic eye disease associated with diabetic retinopathy (DR), diabetic macular edema and glaucoma [1]. Over 90% of patients with type 1 diabetes and around 60% of patients with type 2 diabetes will suffer from a level of DR ranging from blurred vision to complete vision loss [2]. Given the increasing incidence of diabetes, there is thus a significant need to understand the mechanisms which regulate DR, with the aim of developing effective therapeutic agents for the complication.

Endothelial cell injury is a hallmark of the microvascular complications observed due to chronic hyperglycaemia in diabetes. Indeed, small improvements in glycosylated-haemoglobin directly correlate with a significantly reduced risk of developing DR [3]. Hyperglycaemia increases metabolic disruption, inflammation and hypoxia in patients with diabetes, associated with the pathophysiology of DR [2]. DR, characterised by retinal lesions, results in morphological changes and increases in vascular permeability of the retinal microvasculature [4]. In non-proliferative retinopathy, this permeability causes macular edema which, over time, leads to capillary widening and degeneration of the retina whereas in proliferative retinopathy, there is excessive formation of blood vessels (vascularisation) around the optic disk; this aberrant neovascularisation is a major contributor to vision loss and has been attributed to hyperglycaemia-induced increase in local vitreous vascular endothelial growth factor (VEGF) levels in patients with diabetes [5–8]. Indeed, current treatment for patients with non-proliferative DR utilises anti-VEGF agents to reduce macular edema and improve outcomes for patients [4, 9]. However, there are concerns regarding the safety of anti-VEGF agents over the long term, with a small number of cases indicating side-effects such as an increased risk of neurodegeneration for the remaining healthy retina [10, 11]. There is thus a need to investigate potential alternatives to current anti-VEGF agents to improve outcomes for patients with DR.

We have previously demonstrated a role for the commonly-consumed artificial sweetener, sucralose, in attenuating VEGF-induced vascular leak in the pulmonary endothelium [12]. These studies indicate that, through binding to the sweet taste receptor T1R3, sucralose tightens the microvascular endothelium and protects the barrier against leak from several agonists including the bacterial endotoxin LPS. Whilst these studies indicate a protective effect of the sweet taste sensing pathway in reducing leak at the pulmonary microvasculature, a role for artificial sweeteners in regulating the retinal endothelium has not been previously studied.

Sweet taste sensing is mediated by the sweet taste receptors T1R2 and T1R3 which form a heterodimer; however, T1R3 is also able to form a homodimer [13]. These G protein coupled receptors (GPCRs) are stimulated by low concentrations of acutely sweet molecules such as artificial sweeteners (< 1 mM) or exceedingly high concentrations of glucose (> 300 mM) [14]. We previously demonstrated that sucralose regulates expression and phosphorylation of key signalling molecules such as p110α-PI3K, MLC2 and Src in the pulmonary endothelium [12]. Interestingly, these signalling molecules are all linked to processes associated with neovascularisation, including endothelial cell migration, adhesion, contraction and tube formation [15–17]. Therefore, we hypothesised that T1R2 and T1R3 signalling, activated by sucralose, would have an effect on vasculogenic potential of the retinal endothelium.

In the present study we investigate, for the first time, the role of the sweet taste receptor in regulating the retinal endothelium. We demonstrate that activation of the sweet taste receptor, with the artificial sweetener sucralose, attenuates VEGF-induced leak across the retinal endothelial barrier. We further demonstrate that VEGF mediates excessive cell migration, adhesion, proliferation and tube formation in a retinal microvascular endothelial cell line which is blocked by sucralose. Interestingly, the protective effect of sucralose is mediated through the sweet taste receptor T1R3 but not T1R2. Finally, we demonstrate a role for Akt as a key signalling molecule downstream of T1R3 which regulates the protective effect of sucralose. Our studies show that sweet taste sensing through T1R3 plays a significant role in aberrant vascularisation processes which are seen in diabetes and highlights a potential anti-VEGF therapeutic agent for patients with DR.

## Methods

### Cell lines, reagents and ethics

Human retina (RMVEC) and lung (LMVEC) microvascular endothelial cells were purchased from Cell Systems (Kirkland, WA) and ATCC (Teddington, UK) respectively. RMVEC and LMVEC were cultured in vascular cell media supplemented with culture boost or endothelial cell BBE kit, respectively. Endothelial cells were used between passage 2 and 9 and maintained the traditional endothelial cell characteristics of von Willebrand factor and vascular endothelial (VE)-cadherin expression, uptake of acetylated LDL, and positive staining for the lectin *Griffonia simplicifolia*.

TRIzol, SuperscriptII and recombinant human VEGF protein were purchased from ThermoFisher (Paisley, UK). siRNA and DharmaFECT™ reagent were obtained from Dharmacon (Cambridge, UK). Antibodies directed against T1R2, T1R3, VEGFR2 (Flk-1), phosphorylated (Ser^473^) and total Akt1/2, and actin were purchased from Santa Cruz Biotechnology (Santa Cruz, CA). Antibodies were selected from validation studies (https://scicrunch.org/resources). Matrigel Basement Membrane Matrix was obtained from BD Biosciences (Oxford, UK). All other reagents were purchased from Sigma Aldrich (Dorset, UK).

All experimental protocols were approved by the Departmental Research Ethics Panel at Anglia Ruskin University prior to start.

### Transient transfection

RMVEC were transiently transfected with T1R2 or T1R3 SMARTpool siGENOME siRNA duplexes (300 nM), or non-specific, scrambled duplexes, using the Dharmafect™ reagent 4, as per the manufacturer’s guidelines. At 42 h following transfection, cells were exposed to sucralose (0.1 mM) in the presence or absence of zinc sulphate (0.7 mM), VEGF (100 ng/ml) or SC79 (10 μM) for 6 h. H_2_O was used as a vehicle for all treatments except SC79, where ethanol was used. Confirmation of knockdown was performed using RT-PCR and Western blotting analysis of mRNA and protein expression respectively.

### RT-PCR

Total RNAs were extracted from LMVEC and RMVEC using the TRIzol reagent as per the manufacturer’s instructions. RNA was purified using the acid phenol/chloroform system and reverse transcribed using SuperScriptII. T1R2 and T1R3 transcripts were measured with human β-actin primers (forward primer CACCAACTGGGACGACAT; reverse primer ACAGCCTGGATAGCAACG) used as the house-keeping gene as described previously [18]. Expression of the *Tas1r2* and *Tas1r3* gene was measured using specific intron-spanning primers (T1R2: forward primer AATGTCCAGCCGGTGCTCTA, reverse primer CATCGCTGATGGCGCTGTA; T1R3: forward primer TTCCCCCAGTACGTGAAGAC, reverse primer CAGAGAACGTCTGGTGGTGA). Relative gene expression level was analysed, for each sample, using the ΔCt method where ΔCt = (Ct_Tas1r_ − Ct_β-actin_) corresponding to the detected threshold cycles for the target gene and β-actin control.

### Western blotting

LMVEC and RMVEC were lysed with RIPA buffer, resuspended in Laemmli buffer (50 μg) and subjected to immunoblot analysis. Immunoblot analyses were performed on 10% SDS-PAGE using primary antibodies specific to T1R2, T1R3, phosphorylated Akt1/2 (Akt^S473^), total Akt1/2 and β-actin at a dilution of 1:1000, except actin (1:5000), and secondary antibody at dilutions of 1:5000. Densitometry was performed using gel analysis software on ImageJ.

### Whole cell ELISA

RMVEC were transiently transfected with siRNA for 42 h, followed by exposure to sucralose and VEGF for a further 6 h. Cells were then rinsed once with DPBS and fixed using 1% paraformaldehyde at room temperature for 10 min. Whole cell ELISA was then performed as previously described [12, 19] using antibodies specific to the extracellular domain of T1R2 (H-90, αα 201-390), T1R3 (G-2, αα 320-499) and VEGFR2 (Flk-1, Q-20, αα unspecified), and fluorescent-conjugated secondary antibodies measured at 1 s exposure time using a florescent plate reader (Victor, Perkin Elmer).

### Endothelial monolayer permeability

Endothelial monolayer permeability was assessed using the FITC-dextran permeability assay and validated with TER (EVOM^2^; World Precision Instruments, Herts, UK). For analysis of monolayer permeability, RMVEC were transiently transfected with siRNA for 42 h on collagen-coated Transwell filters followed by exposure to sucralose, VEGF and zinc sulphate. Addition of treatments was made at the same time and permeability was measured at 6 h following treatment. FITC-conjugated to 40 kDa dextran was added to media in the upper chamber of the Transwell filter, allowed to equilibrate for 360 s at 37 °C, and a sample (100 μl) of media from the lower chamber was collected and analysed at 488 nm using a fluorescent plate reader (Victor, Perkin Elmer). Permeability (%) was calculated by fluorescence accumulated in the lower chamber divided by fluorescence in the upper chamber, multiplied by 100.

### Cell viability assay

RMVEC were transiently transfected with siRNA for 42 h, followed by exposure to sucralose for a further 6 h. Viability was assessed using the Cell Counting Kit-8 (CCK-8) as per the manufacturer’s guidelines with absorbance read at 450 nm using a microplate reader (Tecan Sunrise). Viability was calculated as % normalised to vehicle.

### Cell proliferation assay

RMVEC were transiently transfected with siRNA for 24 h, and then quiesced in media with 1% FBS for a further 18 h. Cells were exposed to sucralose, VEGF, zinc sulphate and SC79, prepared in media with 1% FBS, for 6 h and counted using a haemocytometer.

### Cell migration assay

RMVEC were transiently transfected with siRNA for 42 h, and scratched using a pipette tip and immediately treated with sucralose, VEGF, zinc sulphate and SC79 for a further 6 h. Cell migration was monitored at 2 h time intervals following the initial scratch and images were captured at × 10 magnification using a Zoe™ Cell Imager (BioRad). Cell migration was assessed using the MiToBo analyser software in Image J as previously described [20], with an average was assessed from 2 wells to represent an n of 1.

### Cell adhesion assay

RMVEC were transiently transfected with siRNA for 46 h, and then replated and immediately exposed to sucralose, VEGF, zinc sulphate and SC79 for a further 2 h. Cells were then rinsed once with DPBS and the CCK-8 kit was used (as described in ‘Cell viability assay’) to quantify adherent cells.

### In vitro tube formation

Transfected RMVEC were plated directly onto Matrigel™-coated wells for 42 h at 37 °C. Cells were then exposed to sucralose, VEGF, zinc sulphate and SC79 for a further 6 h. Images of tube formation were captured at × 10 magnification using a Zoe™ Cell Imager (BioRad). The number of joints and tubes were calculated by using the Angiogenesis Analyser software in Image J as previously described [20]. An average from two wells was assessed to represent an n of 1.

### Statistical analysis

The experimental number is presented in the legend for each experiment. For two groups, the variance in data sets was analysed using the Mann–Whitney test followed by the appropriate *t* test. For three or more groups, variance was assessed by using Bartlett’s test with data sets not reaching significance studied by Kruskal–Wallis test followed by Dunn’s test. For all other data sets, differences among the means were tested for significance in all experiments by ANOVA with Tukey’s range significance difference test. Significance was reached when *p* < 0.05. Values are presented as mean ± standard error mean (S.E.M.)

## Results

### Expression of the sweet taste receptors, T1R2 and T1R3, in a human retinal microvascular endothelial cell line

**Fig. 1:**
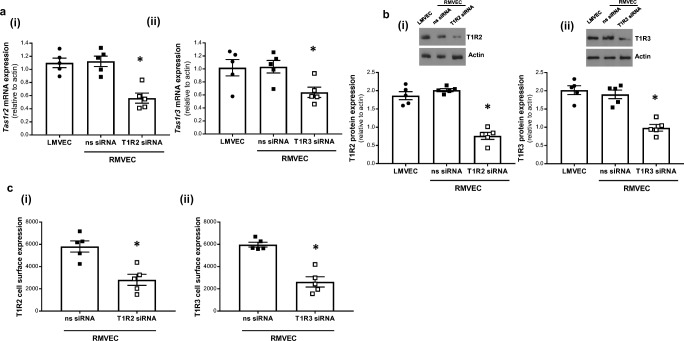
Expression of the sweet taste receptors, T1R2 and T1R3, in retinal microvascular endothelial cells. mRNA (*panel a*) and protein expression (*panel b and c*) of the T1R2 (i) and T1R3 (ii) gene and protein in retinal microvascular endothelial cells (RMVEC) and positive control lung microvascular endothelial cells (LMVEC). siRNA knockdown of T1R2 and T1R3 was performed in RMVEC to validate expression. Gene and protein expression is analysed relative to β-actin. A representative blot is included for Western blotting (*inset*, *panel b*). Cell surface expression (*panel c*) of T1R2 (i) and T1R3 (ii) was determined with whole cell ELISA in retinal microvascular endothelial cells. *n* = 5. Data is expressed as mean ± S.E.M. *p < 0.05 versus ns siRNA

The sweet taste receptors, T1R2 and T1R3, form a heterodimeric complex (T1R2/T1R3) or T1R3 forms a homodimeric complex (T1R3/T1R3) for sweet taste sensing [13]. We have previously demonstrated mRNA and protein expression of T1R3 in the pulmonary endothelium, at levels similar to that of the small intestine [12]. Here we demonstrate that mRNA and protein expression levels of both T1R2 and T1R3 in RMVEC were comparable to expression in pulmonary endothelial cells (LMVEC) (Fig. 1a, b). T1R2 and T1R3 are G protein coupled receptors which function at the plasma membrane [13] therefore we next assessed localisation of the receptors to the cell surface. Using extracellular antibodies and whole cell ELISA, expression of both T1R2 and T1R3 was demonstrated at the cell surface of RMVEC (Fig. 1c). To confirm specificity of primers and antibodies, siRNA knockdown of each receptor was performed. The expression of mRNA, whole cell and cell surface protein was significantly reduced, to a similar degree, following knockdown of T1R2 and T1R3 (Fig. 1a–c). These studies demonstrate, for the first time, that the sweet taste receptors, T1R2 and T1R3, are expressed in a human retinal microvascular endothelial cell model.

### Artificial sweetener sucralose attenuates VEGF-induced vasculogenic processes in a retinal microvascular endothelial cell model

**Fig. 2:**
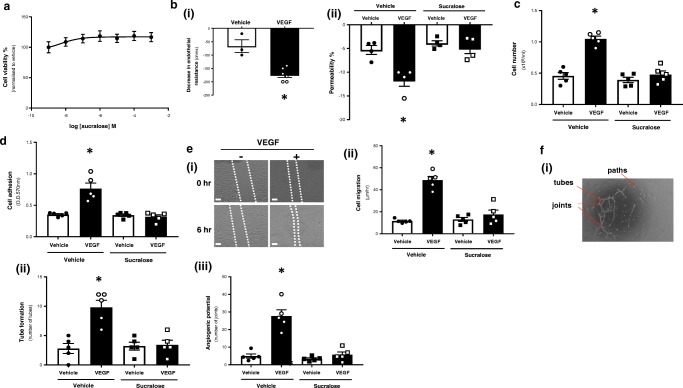
An agonist for sweet taste receptors T1R2 and T1R3, sucralose, attenuates VEGF-induced angiogenic processes in the retinal microvascular endothelium. *Panel a*: cell viability of RMVEC was measured by CCK8 assay following exposure to sucralose (1 nM–1 mM). *Panel b–f*: changes in retinal endothelial cell: monolayer permeability (*panel b*), proliferation (*panel c*), adhesion (*panel d*), migration (*panel e*), tube formation (*panel f* (*ii*)) and angiogenic potential (*panel f* (*iii*)) were measured following exposure to sucralose (0.1 mM) in the presence (closed bars) and absence (open bars) of VEGF (100 ng/ml). Validation of FITC-dextran permeability assay is shown using TER with VEGF (*panel b* (*i*)). A representative image of the wound-healing assay (*panel e* (*i*)) and Matrigel™ assay (*panel f* (*i*)) is shown. *n* = 5. Data is expressed as mean ± S.E.M. *p < 0.05 versus vehicle for VEGF

We next sought to establish whether activation of T1R2 and T1R3, by low concentrations of acutely sweet molecules [21], exhibited an effect on retinal endothelial cell function. As sucralose did not exert any effect on RMVEC viability (Fig. 2a), the artificial sweetener was utilised at the concentration previously identified to be protective against endotoxin-induced barrier disruption in the pulmonary endothelium (0.1 mM) [12]. We next sought to establish whether sucralose was protective against VEGF-induced permeability in the cell model of the retinal endothelium. Exposure of RMVEC to VEGF significantly decreased monolayer resistance and increased permeability as measured by TER and FITC-dextran permeability assay respectively (Fig. 2b); however, VEGF-induced permeability was significantly attenuated by sucralose (Fig. 2b (ii)). Whilst permeability is an indicator of non-proliferative DR, a key pathophysiology of proliferative DR is aberrant vasculogenesis of the retinal microvasculature [6]. Therefore, we next studied the effect of sucralose on vasculogenic processes in the retinal endothelium. RMVEC exposed to sucralose alone, in the absence of VEGF, exhibited no change in cell proliferation, adhesion, migration or tube formation (Fig. 2c–f). RMVEC exposed to VEGF displayed a significant increase in proliferation, adhesion and migration, which was blocked by sucralose (Fig. 2c–e). Likewise, VEGF-induced tube formation and angiogenic potential, denoted as number of joints, in RMVEC was significantly attenuated by exposure to sucralose (Fig. 2f). These studies demonstrate that activation of the sweet taste receptors, T1R2 and T1R3, exerts a protective effect on the retinal endothelial cell model, to blunt VEGF-induced vasculogenic processes. Similar to VEGF, exposure of the endothelium to high glucose concentrations has been demonstrated to increase vasculogenic processes in the endothelium, linked to DR [22]. Exposure of RMVEC to sucralose significantly reduced high glucose-induced cell migration and proliferation (data not shown), indicating a pan-protective role of sucralose on the retinal endothelium.

### Sucralose attenuates VEGF-induced vasculogenic processes in retinal microvascular endothelial cells through the sweet taste receptor T1R3

**Fig. 3:**
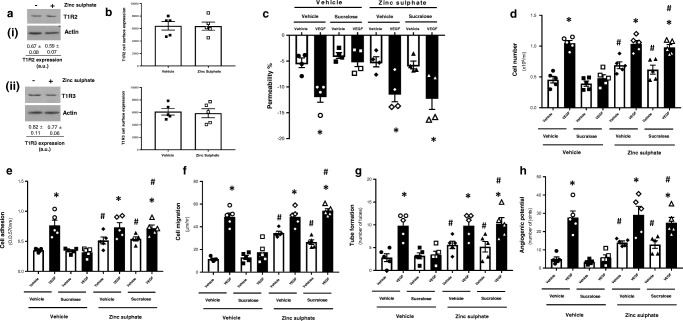
Inhibition of the sweet taste receptors T1R2 and T1R3, through zinc sulphate, blocks the protective effect of sucralose on VEGF-induced angiogenic processes in the retinal microvascular endothelium. Retinal microvascular endothelial cells were exposed to the sweet taste receptor inhibitor, zinc sulphate (0.7 mM), in the presence and absence of sucralose (0.1 mM) and VEGF (100 ng/ml). *Panel a*: protein expression of T1R2 (*i*) *and T1R3* (*ii*) was assessed by Western blotting of RMVEC lysates. *Panel b*: cell surface expression of T1R2 (*i*) *and T1R3* (*ii*) was determined by whole cell ELISA in retinal microvascular endothelial cells. *Panel c–h*: changes in retinal endothelial cell: monolayer permeability (*panel c*), proliferation (*panel d*), adhesion (*panel e*), migration (*panel f*), tube formation (*panel g)* and angiogenic potential (*panel h*) were measured following exposure to sucralose (0.1 mM) in the presence (closed bars) and absence (open bars) of VEGF (100 ng/ml). *n* = 5. Data is expressed as mean ± S.E.M. *p < 0.05 versus vehicle for VEGF, #*p* < 0.05 versus vehicle for zinc sulphate

**Fig. 4:**
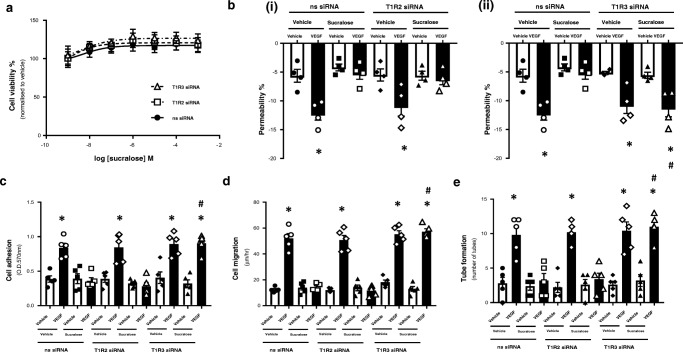
Inhibition of T1R3, but not T1R2, blocks the protective effect of sucralose on VEGF-induced angiogenesis in the retinal microvascular endothelium. RMVEC were transiently transfected with T1R2 or T1R3 siRNA, or a non-specific scrambled siRNA (ns) for 24 h prior to treatment with sucralose (0.1 mM) and VEGF (100 ng/ml). *Panel a*: cell viability of RMVEC was measured by CCK8 assay following exposure to sucralose (1 nM–1 mM). *Panel b*: changes in retinal endothelial cell monolayer permeability were determined using the FITC-dextran permeability assay in RMVEC with T1R2 (*i*) or T1R3 (*ii*) siRNA knockdown. *Panel c–e*: changes in retinal endothelial cell: adhesion (*panel c*), migration (*panel d*), tube formation (*panel e)* were measured following exposure to sucralose (0.1 mM) in the presence (closed bars) and absence (open bars) of VEGF (100 ng/ml). *n* = 5. Data is expressed as mean ± S.E.M. *p < 0.05 versus vehicle for VEGF, #*p* < 0.05 versus ns siRNA

To study whether sucralose acts on retinal endothelial cells through the sweet taste receptors, T1R2 and T1R3, the next experiments were performed with inhibition of the sweet taste receptors. Zinc sulphate, a chemical inhibitor of sweet taste receptor activity, with no impact on sweet taste receptor whole cell or cell surface expression (Fig. 3a, b) was utilised [23, 24]. RMVEC were exposed to zinc sulphate in the presence and absence of VEGF and sucralose and vasculogenic processes were assessed. The protective effect of sucralose, in attenuating VEGF-induced permeability and vasculogenic processes in RMVEC, was blunted by zinc sulphate (Fig. 3d–h). Interestingly, in the absence of VEGF, zinc sulphate had no impact on endothelial monolayer permeability (Fig. 3c); however, cell proliferation, adhesion, migration, tube formation and angiogenic potential were all significantly increased by zinc sulphate (Fig. 3d–h). These studies indicate that chemical inhibition of T1R2 and T1R3 blocks the protective role of sucralose in the retinal microvascular cell line. To establish the role of each receptor in regulating this protective effect, we next used siRNA to perform molecular inhibition of T1R2 and T1R3. Knockdown of T1R2 or T1R3, confirmed by reduced mRNA and protein expression (Fig. 1), had no impact on RMVEC viability compared to non-specific siRNA control (Fig. 4a). Following knockdown of T1R2, sucralose significantly reduced VEGF-induced permeability of the retinal endothelial barrier, similar to the effect in cells transfected with non-specific siRNA (Fig. 4b (i)). Conversely, following molecular inhibition of T1R3 in RMVEC, sucralose had no effect on VEGF-induced barrier disruption (Fig. 4b (ii)). Likewise, sucralose significantly attenuated VEGF-induced endothelial cell adhesion, migration and tube formation in RMVEC with T1R2, but not T1R3, knockdown (Fig. 4c–e). These data demonstrate that the protective role of sucralose, in blocking VEGF-mediated vasculogenic processes in retinal endothelial cells, is mediated through the sweet taste receptor, T1R3.

### Sucralose attenuates VEGF-induced vasculogenic processes in the retinal microvascular endothelial cell line by inhibiting Akt activity

**Fig. 5:**
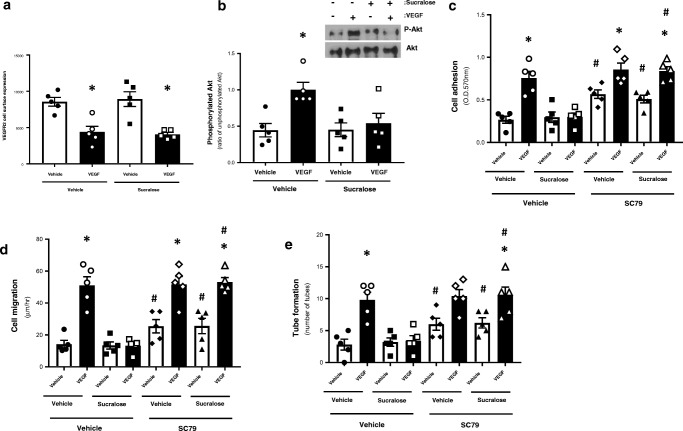
Sucralose regulates VEGF-induced angiogenesis in the retinal endothelium through suppressing Akt activity. *Panel a and b*: RMVEC were exposed to sucralose (0.1 mM) in the presence and absence of VEGF (100 ng/ml). VEGFR2 cell surface expression (*panel a*) and Akt phosphorylation (*panel b*) were measured using whole-cell ELISA and Western blotting respectively. A representative blot is shown. *Panel c–e*: RMVEC were exposed to the Akt activator, SC79 (10 μM) or vehicle (ethanol), followed by treatment with sucralose (0.1 mM) in the presence and absence of VEGF (100 ng/ml). Changes in retinal endothelial cell: adhesion (*panel c*), migration (*panel d*) and tube formation (*panel e*) were measured following exposure to sucralose (0.1 mM) in the presence (closed bars) and absence (open bars) of VEGF (100 ng/ml). *n* = 5. Data is expressed as mean ± S.E.M. *p < 0.05 versus vehicle for VEGF, #*p* < 0.05 versus vehicle for SC79

Finally, we sought to understand the mechanism through which T1R3 regulates VEGF-mediated vasculogenic processes in the retinal microvasculature. VEGFR2 is a major regulator of vasculogenesis [25, 26] therefore we studied whether sucralose exerted a direct effect on expression of the receptor at the cell surface. As previously described [27], RMVEC exposed to VEGF displayed a significant decrease in VEGFR2 expression at the cell surface (Fig. 5a). Interestingly, VEGFR2 surface expression was not affected by sucralose (Fig. 5a). VEGF-VEGFR2 binding triggers the PI3K/Akt signalling pathway which plays a key role in vasculogenic processes therefore we next sought to assess whether sucralose exerts a protective role on the retinal endothelium through Akt signalling [28, 29]. Activity of Akt1/2, assessed by Western blot analysis of serine 473 [28], was significantly increased in RMVEC exposed to VEGF (Fig. 5b). Interestingly, sucralose had no effect on Akt activity in the absence of VEGF but the sweetener attenuated VEGF-induced Akt phosphorylation (Fig. 5b). To establish whether sucralose protects against VEGF-mediated vasculogenesis through inhibition of Akt, RMVEC were exposed to the Akt activator, SC79, in the presence and absence of VEGF and sucralose. SC79 alone significantly increased cell adhesion, migration and tube formation in retinal endothelial cells (Fig. 5c–e). Following exposure to SC79, VEGF significantly increased permeability (Supplementary Table 1) and vasculogenic processes in RMVEC in the presence of sucralose (Fig. 5c–e). Activation of Akt was thus able to block the protective effect of sucralose and restore VEGF-induced vasculogenesis in the cell model of the retinal endothelium.

Taken together, these date demonstrate that activation of the sweet taste receptor, T1R3, by the artificial sweetener sucralose, protects a retinal microvascular endothelial cell line against VEGF-induced vasculogenesis. We further show that sucralose exerts this protective effect by blocking Akt activation, indicating that the T1R3 represents a novel potential therapeutic target for reducing vascular permeability and vasculogenesis in patients with non-proliferative and proliferative DR.

## Discussion

In this study, we present findings which demonstrate, for the first time, the expression of sweet taste receptors T1R2 and T1R3 in a cell model of the human retinal microvascular endothelium. Our research identifies a role for sweet taste sensing in regulating the retinal endothelium; activation of the sweet taste receptor with sucralose protects the retinal endothelium against VEGF-induced permeability and VEGF-induced vasculogenic processes, linked to non-proliferative and proliferative DR respectively. We further demonstrate that the protective effect of sucralose is mediated through the receptor T1R3, and not T1R2, and by blunting VEGF-induced Akt activity in retinal endothelial cells. Findings from the study demonstrate a novel mechanism through which the retinal endothelium is regulated and indicate a potential therapeutic intervention to reduce the aberrant retinal vasculogenesis observed in patients with DR.

Hyperglycaemia in diabetes is associated with the aberrant vasculogenesis observed in patients with DR [2]. It may therefore appear counter-intuitive for a sweet taste receptor to exert a protective effect on the endothelium; however, glucose concentrations of ~ 300 mM are needed to stimulate T1R2 and T1R3, concentrations which are not physiological in the circulation [30]. In contrast, sucralose, like many artificial sweeteners, stimulates the sweet taste receptor at low concentrations (< 1 mM) [14]. Consumption of artificial sweeteners has increased in the last 15 years with 62% of all soft drinks consumed being sweetened by these non-nutritive sweeteners [31]. In addition to being recommended for patients with diabetes to reduce intake of sugar, artificial sweeteners are also used as an aid in weight-loss/management. There has, however, been significant controversy in recent years surrounding the use of artificial sweeteners in the diet with both positive and negative impacts on appetite, hunger, weight gain and predisposition for glucose intolerance. As such, the majority of these studies have focused on the small intestine environment, gut microbiota and incretin signalling. These studies suggest increased sweeteners in the diet correlate with weight gain and incidence of diabetes [32]. Following consumption in the diet, a significant proportion of artificial sweetener is absorbed by the small intestine into the systemic circulation and, with the exception of aspartame, is excreted in a largely unmetabolised form [33, 34]. However, there is still a limited understanding of the physiological relevance of T1R2 and T1R3 in the vasculature, and the resulting impact of sweeteners once they are in the circulation.

The blood-retinal barrier forms a selectively-permeable filter maintained by junctional complexes in retinal endothelial cells to form the inner barrier [35]. The pathophysiology of DR is based on multiple components, such as loss of pericyte function and release of inflammatory cytokines by the retinal pigmented epithelium; however, permeability of the retinal endothelial monolayer, via elevated VEGF signalling, is a key pathophysiology of macula edema observed in patients with non-proliferative DR [2, 4]. We have previously demonstrated a role for sweet taste sensing in reducing vascular leak in the lung microvasculature linked to endotoxin-induced pulmonary edema [12]. In the present study, we observe that activation of T1R3, with the artificial sweetener sucralose, exerts a similar effect in protecting the human retinal endothelial monolayer from VEGF-induced permeability. These findings therefore demonstrate a pan-protective role of the receptor which is irrespective of injury stimulus or vascular bed. Whilst these studies indicate the protective effect of a T1R3 agonist in a human cell model of the retinal microvasculature, further studies are needed to confirm the physiological relevance of these findings in vivo.

In patients with proliferative DR, aberrant vasculogenic processes such as hyperproliferation and excessive neovascularisation, are observed which are, in part, mediated through pro-angiogenic VEGF signals [5–8]. We and others have previously demonstrated a close association between mechanisms which regulate permeability of the endothelial barrier and vasculogenic processes [16, 19, 20]. Therefore, in the present study, we assessed the effect of T1R3 stimulation on vasculogenic processes in a cell model of the retinal endotheliums, such as proliferation and tube formation. Activation of the sweet taste receptor by sucralose exerted a protective effect on VEGF-mediated vasculogenic processes such as excessive proliferation, adhesion, migration and tube formation. Interestingly, this protection was mediated through the sweet taste receptor T1R3 rather than T1R2. Whilst the sweet taste receptor is typically considered to be a heterodimer of T1R2/T1R3, Nelson et al. have previously demonstrated that T1R3, but not T1R2, can form a homodimer for sweet taste sensing [13, 14]. It is therefore likely that sucralose binds to the T1R3 homodimer to protect the retinal vasculature against VEGF-induced vasculogenesis. Given that human retinal microvascular endothelial cells express T1R2 mRNA and protein at levels similar to T1R3, yet T1R2 is not involved in permeability or vasculogenesis, this raises interesting questions regarding the potential role for this GPCR in the microvasculature.

Akt has been implicated as a key signalling molecule through which VEGF regulates vasculogenic processes [36]. VEGF-VEGFR2 binding results in PI3K-dependent phosphorylation and activation of Akt resulting in increased endothelial cell proliferation, migration and permeability [37]. Interestingly, artificial sweeteners have been demonstrated to have opposing effects on Akt phosphorylation, depending on the differentiation stage of the cell and the phosphorylation site investigated [38, 39]. In the present study, we demonstrated that sucralose blocked VEGF-induced phosphorylation of Akt at Ser^473^. Given the role of Akt phosphorylation at this residue in regulating FOXO1 target genes associated with vasculogenesis [40, 41], our data indicates that T1R3 activation blocks vasculogenic processes through Akt dephosphorylation. Indeed, we observed that chemical activation of Akt reversed the protective effects of sucralose on vasculogenesis. Sucralose had no impact on Akt phosphorylation, or vasculogenic processes, in the healthy endothelium (in the absence of VEGF). Therefore it is possible that T1R3 signalling downregulates a specific kinase which is upregulated by VEGF signalling, such as mTOR [37]; however, further studies are needed to fully understand the molecular mechanism through which T1R3 regulates Akt activity in the retinal endothelium.

At present, treatment for patients with non-proliferative and proliferative DR is based on intravitreous injection of anti-VEGF agents, such as ranibizumab, and panretinal laser photocoagulation therapy [42]. Whilst these treatments are effective in reducing retinopathy in patients, long-term use of anti-VEGF agents has been linked to eye inflammation and capillary regression of the remaining healthy microvasculature [43] Given the increasing number of patient with diabetes, and the high chance of these patients developing retinopathy [1, 2], there is a significant need to consider alternate potential therapeutic approaches to reduce permeability and vasculogenesis in the retinal microvasculature. In the present study, we demonstrate that sucralose blocks VEGF-induced permeability and vasculogenic processes. Interestingly, in the healthy endothelium, in the absence of VEGF, we demonstrate that sucralose has no effect on permeability or vasculogenesis. Our studies therefore indicate that T1R3 represents a novel therapeutic target with potential to reduce permeability and vasculogenesis in the retinal endothelium in patients with both proliferative and non-proliferative DR. Further research is needed to understand the physiological effect of sweeteners on pathogenic events leading to retinopathy, as well as the potential side effects of sweeteners in the vasculature, similar to those seen with current anti-VEGF treatment.

## Electronic supplementary material

Supplementary Table 1 (DOCX 12 kb)
