# Supplementary material for: Activation of the sweet taste receptor T1R3 by sucralose attenuates VEGF-induced vasculogenesis in a cell model of the retinal microvascular endothelium
Source: Graefes Arch Clin Exp Ophthalmol. 2018 Oct 23;257(1):71–81. doi: 10.1007/s00417-018-4157-8 (PMC6323138; doi:10.1007/s00417-018-4157-8)
Supplement: Supplementary file 1 — (DOCX 12 kb) [file 417_2018_4157_MOESM1_ESM.docx]

**Supplementary data**

**Supplementary table 1: Sucralose regulates VEGF-induced permeability in retinal endothelial cells through suppressing Akt activity.** RMVEC were exposed to the Akt activator, SC79 (10 µM) or vehicle (ethanol), followed by treatment with sucralose (0.1 mM) in the presence and absence of VEGF (100 ng/ml). Changes in retinal endothelial cell monolayer permeability were determined using the FITC-dextran permeability assay. n=6. Data is expressed as mean ± S.E.M. *p<0.05 versus vehicle for VEGF.

|  | **Vehicle** | | **SC79** | |
| --- | --- | --- | --- | --- |
|  | **Vehicle** | **Sucralose** | **Vehicle** | **Sucralose** |
| **Vehicle** | 4.99 ± 0.48 | 4.16 ± 0.48 | 4.88 ± 0.55 | 5.27 ± 0.76 |
| **VEGF** | 11.38 ± 0.97* | 4.86 ± 0.65 | 10.83 ± 1.05* | 11.59 ± 1.41* |
